# Tempting food words activate eating simulations

**DOI:** 10.3389/fpsyg.2013.00838

**Published:** 2013-11-15

**Authors:** Esther K. Papies

**Affiliations:** Department of Psychology, Utrecht UniversityUtrecht, Netherlands

**Keywords:** grounded cognition, simulation, feature listing, cognitive psychology, self-control, temptation

## Abstract

This study shows that tempting food words activate simulations of eating the food, including simulations of the taste and texture of the food, simulations of eating situations, and simulations of hedonic enjoyment. In a feature listing task, participants generated features that are typically true of four tempting foods (e.g., *chips*) and four neutral foods (e.g., *rice*). The resulting features were coded as features of eating simulations if they referred to the taste, texture, and temperature of the food (e.g., “crunchy”; “sticky”), to situations of eating the food (e.g., “movie”; “good for Wok dishes”), and to the hedonic experience when eating the food (e.g., “tasty”). Based on the grounded cognition perspective, it was predicted that tempting foods are more likely to be represented in terms of actually eating them, so that participants would list more features referring to eating simulations for tempting than for neutral foods. Confirming this hypothesis, results showed that eating simulation features constituted 53% of the features for tempting food, and 26% of the features for neutral food. Visual features, in contrast, were mentioned more often for neutral foods (45%) than for tempting foods (19%). Exploratory analyses revealed that the proportion of eating simulation features for tempting foods was positively correlated with perceived attractiveness of the foods, and negatively with participants’ dieting concerns, suggesting that eating simulations may depend on individuals’ goals with regard to eating. These findings are discussed with regard to their implications for understanding the processes guiding eating behavior, and for interventions designed to reduce the consumption of attractive, unhealthy food.

## INTRODUCTION

How do people represent food? Although eating food, enjoying food, and increasingly, restricting food intake, play a major role in our lives, we know surprisingly little about how food is represented conceptually. The current research is part of an attempt to answer this question. Specifically, it will be examined whether simulations of eating experiences play a role in our representations of food concepts, particularly for tempting food. This may have important implications for understanding how we regulate our eating behavior, as well as for the development of effective interventions for healthy eating.

Given that food and eating are necessary for our physical survival, the nutrients of food and its effects on the body could be a highly likely way of representing food conceptually. However, food is often selected for its taste and the pleasure of eating it (e.g., Drewnowski, 1995), and thus serves a strong hedonic purpose (e.g., Lowe and Butryn, 2007). In addition, eating certain foods also has important social and cultural functions (see Mintz and Bois, 2002, for a review). Thus, these dimensions might form part of the conceptual representation of food as well. In addition, some foods are eaten habitually in certain emotional states (“comfort foods”; e.g., Wansink et al., 2003; Locher et al., 2005), in certain situations, at specific events, or in certain ways (see van’t Riet et al., 2011). Although we know that contextual cues play an important role in triggering desire and motivated behavior (see Kavanagh et al., 2005), it is unclear whether such eating situations form part of the actual conceptual representation of food. The present paper therefore addresses the question to what degree features related to the taste, the pleasure, and the context of eating play a role in our cognitive representation of food items.

Why is this question relevant? Consider for example a customer who walks through a grocery store and passes the aisle where various kinds of chips are visible on the shelves. When seeing the bags of chips, the representation of chips as being a fried potato product may be activated, along with the nutritional knowledge that the product contains a lot of salt and fat, and thus provides a lot of energy. However, the customer may also activate a much richer representation of the food that is based on earlier eating experiences. In that case, the chips might trigger a memory of their salty and savory taste and their crunchy texture; they might trigger a sense of enjoyment; they might be represented in the background situation of eating them in a sociable setting, for example, with friends while watching a movie on the sofa. To the degree that these re-experiences activate reward signals, they may trigger a desire for chips that may not be activated by the mere knowledge that chips are a fried potato-product with certain nutritional properties (Papies and Barsalou, in preparation; Stroebe et al., 2013; see also Kavanagh et al., 2005). In sum, it is suggested that the conceptual representation that is activated spontaneously in response to a food item may have important implications for eating motivation, and thus for actual behavior. In other words, thinking about an item like chips in terms of enjoying its taste and texture in a relaxed social situation is more likely to trigger consumption, than thinking about it in terms of nutritional properties. Thus, understanding the representation of food, and whether food is represented by earlier eating experiences, is crucial for understanding the development of desire for foods, and for developing effective ways to regulate this in order to facilitate healthy eating behavior.

### A GROUNDED COGNITION PERSPECTIVE ON FOOD

Research in the domain of grounded cognition suggests that our knowledge about objects is represented by partial reenactments, or simulations, of earlier perceptual experiences in the relevant modalities (e.g., Barsalou, 2008,^2009^). For example, simply thinking about a stimulus, such as a cup or a hammer, activates similar brain areas as when processing the stimulus perceptually or when actually using it, which prepares us for effectively interacting with it (Martin et al., 1996; Tucker and Ellis, 2001; Pulvermüller and Fadiga, 2010). Similarly, when confronted with the emotional expression of another person, we spontaneously simulate their respective emotional state in ourselves, which facilitates social understanding and interaction (Barsalou et al., 2003; Niedenthal, 2007). Indeed, an important assumption underlying the grounded cognition account is that the simulations that underlie our knowledge of the world support goal-directed behavior. In other words, the simulations triggered by external stimuli tell us what we can do with them, how to do it, and what may result, so that we are prepared to engage with the respective objects in a way that supports our goals (Barsalou, 2009).

Our simulations of concepts typically also include background settings and events (Barsalou, 2009). In other words, when thinking about an object, we do not activate a simulation of this object in isolation, but in a relevant background situation. Indeed, object nouns have been shown to automatically activate information about locations where these objects are typically found (Borghi et al., 2005). Similarly, when listing typical features of non-present objects (e.g., “car”), participants not only describe perceptual features of the object (“headlight”; “stinks”), but also features of its use (“faster than walking”) and relevant events and situations for using it (“on the highway,” “a long drive”; Wu and Barsalou, 2009).

Applying this grounded cognition account to the representation of food, it follows that food objects may also activate such situated simulations, and more specifically, simulations of eating the food. In addition, and based on the assumption that simulations support goal-directed action, it further follows that attractive foods should be more likely to activate eating simulations than neutral foods. Because eating attractive foods typically leads to more rewarding experiences than eating neutral foods, attractive foods should be more likely to initiate eating simulations, given that the greater anticipated reward motivates doing so.

Which foods, however, are perceived as attractive, and therefore as particularly eating-relevant? Although people’s tastes for specific foods vary widely, humans have evolved to share a strong liking for food that is high in calories, particularly from fat and sugar (Pinel et al., 2000). Such food items are often unhealthy and can interfere with one’s weight regulation goals, so that they are often called food temptations (i.e., attractive but unhealthy). At the same time, they are more likely to attract attention (e.g., Papies et al., 2008; Tapper et al., 2010; Van Dillen et al., 2013), to activate reward-processing areas in the brain (van der Laan et al., 2011), and to trigger spontaneous approach reactions (Papies et al., 2012; Veling et al., 2012). In other words, attractive foods easily trigger goal-directed action toward consuming them, despite being unhealthy. Here, it is predicted that this will also be reflected in their conceptual representations, such that tempting foods are more likely to be represented in terms of eating simulations than neutral foods.

Initial evidence from cognitive and neuroimaging work suggests that eating simulations could indeed play a role in the representation of food. Neuroimaging studies show, for example, that seeing a food item or reading food words activates gustatory and reward areas in the brain, suggesting that perceivers processes the food similarly to as if they were actually eating and enjoying it (e.g., Simmons et al., 2005; Barrós-Loscertales et al., 2012). Similarly, behavioral research in which participants were asked to generate properties typically true of a large number of concepts, including food, showed that participants spontaneously refer to experiences of eating the food, such as “eaten with ice cream” for *cake*, “eaten by peeling” for *banana*, or “is juicy” for *nectarine* (McRae et al., 2005). Finally, in studies asking participants to categorize food (Ross and Murphy, 1999), participants often used so-called script categories, such as “breakfast food,” or “foods you eat with a spoon.” The use of this type of category suggests that food concepts are understood in terms of when and how one interacts with them, thus placing them in a background situation that is relevant to goal-directed action (see also Blake et al., 2007).

In sum, initial evidence suggests that eating simulations might indeed play a role in the representation of food. Yet no research so far has tested systematically whether such eating simulations are activated consistently when participants think of food, and whether this differs for foods that differ in reward value. The research reported here was designed to answer this question.

### THE CURRENT RESEARCH

The feature listing (or property generation) task has been used widely to establish the representation of concepts (e.g., Rosch et al., 1976; Andersen and Cole, 1990; McRae et al., 2005; Wu and Barsalou, 2009; Santos et al., 2011). Thus, the feature listing task was used here to examine the representation of concepts for food. As in a typical feature listing experiment, participants were asked to generate features that are typically true of a concept, and they were asked to do so for a number of both food and non-food words. Although we typically encounter foods as objects rather than as words, food words were used as stimuli here in order to tap into the representation of food concepts in participants’ memory, without providing vivid details from food pictures that could influence the representation they retrieve. In particular, the attractiveness of the food as displayed in the picture could potentially bias the features produced. In addition, when using pictures as stimuli, participants could be describing features of the picture, rather than their representation of the food in memory. To prevent both of these potential problems, food words were used.

For each concept, it is assumed that participants retrieve a specific memory of that concept, of which they then describe a number of features, possibly in various modalities (e.g., visual, auditory, introspection; Wu and Barsalou, 2009). Thus, rather than a static list of features, this paradigm may generate a varied list of features that is heavily idiosyncratic, as it reflects each participant’s recent and/or frequent encounter with a given object. In the context of the current research, this is desirable as it allows participants to provide features of the foods in any way that reflects their idiosyncratic experience and representation of them. The features generated then must be coded systematically in relevant categories to test specific hypotheses of interest.

Again, it is hypothesized that when we are confronted with a food, we spontaneously simulate eating it. Thus, when presented with a food like *chips*, participants in a feature listing task may spontaneously activate and mention features of the food that one experiences when eating it, such as its taste and texture (“crunchy”), the hedonic experience (“tasty”), or a background situation in which chips are typically consumed (“watching a movie”). At the same time, participants could generate category information, such as a specific type of chips (“Lays chips”), they could describe the food visually (“yellow”), or they could refer to health or nutritional properties (“fat”).

Although the current research was mainly designed to test the simulation account of food representations, it is worth noting that alternative accounts potentially exist of how participants produce features for food concepts, for example, one might assume that participants generate features on the basis of word associations (Nelson et al., 2004; Santos et al., 2011). Casual inspection of the Nelson et al., norms found that associations to the food words used in the study to follow consisted largely of category information (“fruit” for the concept *banana,* “orange” for *apple*), as well as forward and backward continuations associated with compound noun phrases (“monster” for *cookie,* “chocolate” for *ice cream*; Nelson et al., 2004). Furthermore, a word association account would not predict the production of eating simulation features, nor that the production of these types of features would differ systematically between tempting and neutral food. Although word associations may well become active as participants generate features of food concepts (see Santos et al., 2011), it is predicted here that participants will, in addition, heavily produce features resulting from eating simulations, particularly for tempting food.

To systematically assess the occurrence of eating simulation features and other features in response to tempting and neutral food, and to distinguish these features from linguistic phrases (e.g., “cookie monster”) and category information, a specialized coding procedure was developed for the current study. In this novel coding procedure, features were coded as part of an eating simulation when they referred to a property of the food object itself that would most likely be experienced when eating the food, such as its taste, texture, and temperature. These sensory features refer to gustatory sensations, as well as to the “mouth feel” of the food. In addition, features that describe (parts of) a background situation in which the food is eaten were also assumed to reflect part of an eating simulation (see Wu and Barsalou, 2009). Finally, the hedonic experience of pleasure or displeasure that one might have when eating the food was assumed to be part of the eating simulation as well. In contrast, features of a situation associated with growing, producing, purchasing, or preparing food were not assumed to be part of an eating simulation, since they are highly salient in situations that do not involve eating the food. Similarly, visual descriptions of food or its parts were not assumed to be part of eating simulations, since although they are clearly perceived during eating, they are also salient in many non-eating situations involving the food. In other words, when participants generate such features, we cannot be sure that they do so because they simulated eating the food. Therefore, they were not treated as part of eating simulations. Finally, category information and features referring to the longer-term health and nutrition implications of eating the food were not assumed to be part of eating simulations. These codings of the features that participants produced allowed for testing the hypotheses that eating simulations represent food in part, and that this is particularly true for tempting food.

## MATERIALS AND METHODS

### PARTICIPANTS

Thirty-three students of Utrecht University (13 men, 19 women, 1 did not provide gender information; mean age 21 years) participated in exchange for a small monetary compensation or course credit.^1^ Participants provided informed consent. The experiment was performed on desktop computers in individual cubicles.

### PROCEDURE, DESIGN, AND MATERIALS

#### Feature listing task

Participants were asked to list features of 16 concepts. The word for each concept was presented on the computer screen individually, and participants typed their answers into an empty text box on the same screen. The critical items of interest were four attractive, but unhealthy foods, namely *vanilla ice cream, cookies, cocktail nuts* (a Dutch snack of peanuts with a fried coating)*,* and *chips* (potato chips from a bag), as well as four neutral, healthy foods, namely *cucumber, apple, banana,* and *rice*. Eight natural kinds and household objects (e.g., *butterfly, phone, mattress*) served as fillers to make the food-related nature of the research less obvious. All items were presented in a different random order for each participant.

Participants were asked to describe properties that are typically true of the object (see McRae et al., 2005; Wu and Barsalou, 2009). They were provided with two examples: the features “heavy, round, cold, gray, can be thrown” for the concept *stone*; the features “yellow, light, rough, handy for doing the dishes” for *sponge*. Participants were encouraged to respond spontaneously, and to write down the typical features that came to mind first. They were asked to write down at least five features, but told that there was room for 15 features.

#### Other measures

After the feature listing task, participants were presented with a number of other measures for exploratory purposes. They were first asked to indicate whether they would currently like to eat each of the eight critical food items used in the feature listing task, mixed in with 25 other foods fillers. Finally, participants completed the restraint scale (Herman and Polivy, 1980), which included two questions about their weight and height (making it possible to compute their body mass index, BMI), along with questions assessing the importance of dieting and being slim as well as dieting success (Fishbach et al., 2003). Participants also indicated their current level of hunger, and when they had last eaten. Finally, they rated 18 food items for their tastiness, including the eight critical foods. After completing the computer tasks, participants were debriefed, paid, and thanked.

### FEATURE CODING SCHEME

A coding scheme was developed for coding the features that participants generated. This was inspired by Wu and Barsalou (2009) coding scheme, but created specifically for the current research in order to test the specific hypotheses developed here. Criteria for coding a feature in a specific category are presented below. All features and how they were coded are provided in **Tables 1 and 2**. A detailed coding manual can be obtained from the author.

**Table 1:** All features generated for the four tempting food items.

Food words	Taste, texture, temperature features	Hedonic features (positive | negative)	Eating situation features	Visual features (including visible parts)	Non-eating situation features	Health features (positive | negative)	Other features (including ingredients)
*Chips*	(very) Salty; paprika (taste); spiced; natural; savory; light; crunch(ing); brittle; hard; rustling; crunchy; crispy	Tasty; delicious; scrumptious	From a bag; for parties; (eaten with a) movie; birthday; with drinks; bowl; to snack; dip sauce; at night; with beer; eat them sociably together; on the sofa	Yellow(ish); round; small; thin; different shapes; red; slices; orange	Frying pan; fried; (packed in a) (plastic) bag; breakable; vulnerable	*(No positive health features)* Fat; unhealthy; gives you pimples	(made of) Potato; edible; snack; (different) varieties; Lays; food
*Vanilla ice– cream*	Sweet; creamy; crème; soft; can be formed; tastes like vanilla; fresh; cold;	(not) Tasty; cooling	For nice weather; scoops; summery; dessert; on a cone; whipped cream; (in) summer; you eat it on vacation; warm; to treat yourself; good with apple pie	Yellow-white; light yellow(ish); whit(ish); round; cream-colored; black dots	(lies in the) Freezer; in a container; sold by the container; ice– cream vendor; from the freezer; you get the best at the Australian; (can) melt(s) (fast); sticky	*(no positive health features)* Unhealthy; (makes you) fat	Dessert; ice cream; vanilla; dairy product; neutral; most popular kind of ice– cream; simple
*Cocktail nuts*	Salty; paprika; savory; savory layer of dough; crunchy; crunching; brittle; peanut (in the middle); crispy; hard; crack a lot when you eat them	Tasty|not tasty	(for) Sociability; drinks; wine; (good with) beer; you eat it around 4 or 5 pm; you drop them often; for parties; end of afternoon; they often sit on the bar; served in a bowl; (when) watching a movie/TV; side dish; bowl; at night	Small; round; brown; sand-colored; gray; orange; different colors; beige; white; colored	Baked; packed; (come in a) bag; grocery store; expensive	*(no positive health features);* Fat; unhealthy; makes you thirsty	Peanuts; tiger nuts *(product name)*; Duyvis *(brand name)*; edible; snack; food, to eat; a lot of exterior; broth
*Cookies*	Savory; crunchy; sweet; hard; breakable; brittle; crispy; crumbly; crumbs; granular; dry	Tasty *(no negative hedonic features)*	Are eaten with tea or coffee; tea; coffee; sociable; (good as a) snack; children love them	Yellow; brown (ish); square; small; round; different shapes; small	(easy to) Bake; baked (in the oven); from the oven; (made of) dough; cookie tin; (you buy them) (in a) pack	*(no positive health features)*unhealthy; bad snack	To eat; (good) snack; chocolate; (different) varieties; a lot, waffles; edible; sugar; raisins; flour; grain; annoying (crumbs); oats; butter; almond shavings

*Note: every feature is listed only once, even if it was mentioned by more than one participant.*

**Table 2:** All features generated for the four neutral food items.

Food words	Taste, texture, temperature features	Hedonic features (positive | negative)	Eating situation features	Visual features (including visible parts)	Non-eating situation features	Health features (positive | negative)	Other features (including ingredients)
*Apple*	(sometimes) Sour; (sometimes) sweet; fresh; juicy; hard, soft; wet; crunchy; fruity; solid	Tasty (*no negative hedonic features*)	Breakfast; in yoghurt; apple pie; to bite; specific smell	(is) round; green; red; yellow, (red or green) peel; (has) core; (has) stem; seeds inside; white inside; several colors; pieces	(Grows on a) tree; caterpillar; toxins; fruit basket; easy to peel; you can make pie of it	Healthy | bad for your teeth	Edible; (a) fruit; Snow White; food; juice; *Dutch proverbs*: “*voor de dorst,” “appeltje te schillen”*
*Banana*	Soft; sweet; fruity; soft flesh; soft inside	Tasty *(no negative hedonic features)*	To peel; good in yoghurt	Yellow; bent; moon; (sits in a) peel; smooth; small; long; (brown spots); long cylinder; sometimes green or brown;	Grows in the tropics; tropical; hard peel; on trees; (toward the) sun; in bundles; import; monkey(s) (eat bananas); grows brown quickly; degradable; ice–cream	Healthy; nutritious; satisfies | constipation	Fruit; nature, edible; food; lazy; no vitamin c; strange; starch
*Rice*	Neutral taste; tasteless; soft; granular; sticky; hard; dry; warm	Tasty|not tasty; annoying; unappetizing	Dish; side dish; nasi; good for dinner; dinner; Chinese food; Asian food; good for wok dishes; good with sate sauce; good for paella	Small; grains; white; grain form; long shape; yellow; brown; transparent	(comes) (from) Asia(n); grown on sawa’s; Chinese; Indonesian; Eastern; (rice) field(s); terraces; packed; cheap; often used in Asian cooking; (has) to cook / to be cooked; can be used to make dessert; large pan; easy; water; to drain; steam; is thrown on weddings; good luck	Healthy; filling; protein; light food (*no negative health features*)	Edible; food; basis; there are several/many kinds; brown rice; nut rice
*Cucumber*	Fresh; fruity; tasteless; juicy; watery; solid; soft inside; hard	Tasty; refreshing *(no negative hedonic features)*	(good in) salad; you eat it cold; slices; children like it	Long (shape); green; round; bent; thin; looks like zucchini; cylinder; straight; wrinkles; peel; row of seeds	Grows on a plant; food for horses; cheap; easy; becomes disgusting when expired; mask	Healthy; vitamins; quenches thirst	Vegetable; (lots of) water; fruit; to eat; not filling; food

*Note: every feature is listed only once, even if it was mentioned by more than one participant.*

#### Taste, texture, and temperature

A feature was coded in this category if it referred to the taste of a food (e.g., “sweet”), its texture (e.g., “crunchy”), or its temperature (e.g., “cold”), as experienced when eating the food.

#### Eating situations

A feature was coded as a situation feature if it referred to an aspect of a situation that involves eating the food. This could be a specific time (e.g., “evening”), place (e.g., “sofa”), or event (e.g., “party”) where the food is eaten; a particular action (e.g., “biting”) or manner of eating (e.g., “from the bag”); an object or utensil used in an eating situation (e.g., “spoon,” “bowl”); another food that you eat with the critical food (e.g., “goes well with chicken”); a specific form that the food can take (e.g., “apple pie”); a prepared dish in which you typically find the food (e.g., “salad”); or a person in an eating situation (e.g., “kids”). In short, features were coded as situation features if they refer to when, where, and how you eat the food, who eats it, and what accompanies eating it.

#### Hedonic features

Features were coded as “hedonic” if they referred to the pleasure or displeasure that can result from eating a food (e.g., “tasty,” “delicious,” “disgusting”). Both positive and negative features were mentioned, and were coded in this category.

Within the hedonic category, features were also coded in two subcategories as either hedonic positive or hedonic negative, thereby enabling a manipulation check of whether the selected foods indeed differed in their anticipated hedonic experience. For the main analysis, however, the overall hedonic category was used, given that both positive and negative hedonic features are assumed to result from eating simulations.

#### Visual features

A feature was coded in this category if it referred to a visible aspect of a food object. This could be the color of the food (e.g., “yellow”), the form it comes in (e.g., “grains”), the form of an individual item (e.g., “round”), visible parts on the outside (e.g., “peel”), or visible parts on the inside (e.g., “seeds”) of a food.

#### Non-eating situations

A feature was coded in this category if it referred to a situation that did not involve eating a food. These features referred to how the food is produced (e.g., “from the oven”), how it is grown (“from a tree”), where it is purchased (e.g., “gelato store”), how it is stored (“tin”), as well as procedures or ingredients needed for making it edible (“steaming”). A feature was also coded into this category if it referred to a non-human agent eating the food (e.g., “monkey” for banana). Features that refer to the temperature or the texture of a food but that are not experienced when eating the food, but that are experienced on other occasions (e.g., during storage or transport) were also coded as non-eating situation features (e.g., “break easily”).

#### Health features

A feature was coded as a health feature if it referred to the health implications of eating a food, or to the food generally being healthy or unhealthy. Examples of features coded for healthy are “healthy,” “nutritious,” or “vitamins.” Examples of features coded for unhealthy are “unhealthy,” “makes you fat,” or “bad for your teeth.”

Within the health category, features were also coded in two subcategories as health positive or health negative, thereby enabling a manipulation check of whether the tasty and neutral foods indeed differed in their perceived healthiness. For the main analysis, however, the overall health category was used.

#### Other features

All other features were coded as belonging to this category. These included category words (e.g., “fruit”), ingredients that the food contains (e.g., “flour”), products that can be derived from the food (e.g., “juice”), or other features that could not otherwise be categorized (e.g., “a lot,” “snow white”).

### CALCULATING PROPORTIONS FOR FEATURE TYPES

The proportion of features that a participant produced in a specific coding category for a given food was calculated by dividing the number of features in the coding category by the number of total features for the food. These proportions were then averaged across the four tempting and the four neutral foods, separately, for each participant. This procedure was followed for all feature types. Then, the proportion of eating simulation features was calculated per participant by adding the proportions of taste, texture, and temperature features, eating situation features, and hedonic features. The proportion of other features was similarly calculated by summing the proportions of the remaining four feature types (visual, non-eating situations, health, and other).

## RESULTS

Participants produced on average 5.16 features per food item (SE = 0.15), which corresponds with the instruction to list at least five features. This number is comparable to one recent study (Santos et al., 2011) but less than in others (Wu and Barsalou, 2009). Fewer features overall were generated for tempting food (*M* = 5.01, SE = 0.16) than for neutral food (*M* = 5.32, SE = 0.15), *F*_(1,32)_ = 8.67, *p* = 0.006, $\eta_{p}^{2}$ =0.21.

All subsequent analyses were conducted on proportions, and means are reported as percentages for ease of interpretation.

**FIGURE 1:**
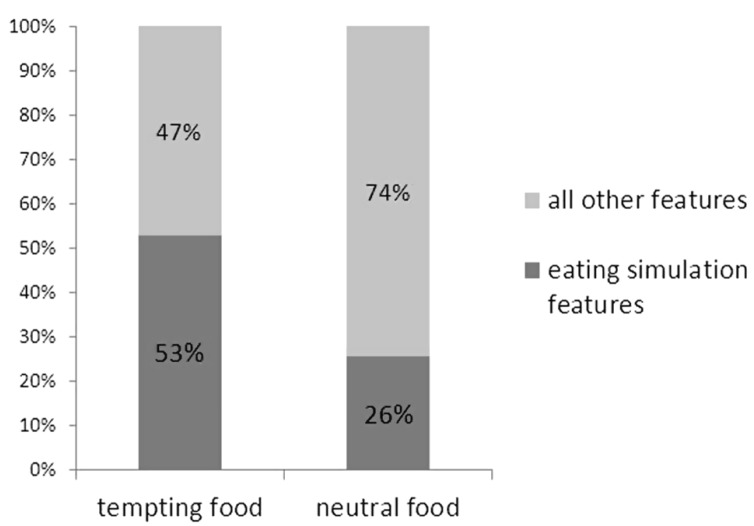
**Percentages of eating simulation features and other types of features for tempting and neutral food.** Eating simulation features are all features in the categories taste, texture, temperature; hedonic; eating situations. Other types of features are all features in the categories visual; non-eating situation; health; other.

To test the hypothesis that participants generate more eating simulation features for tempting than for neutral food, a repeated measures ANOVA was conducted on proportions of eating simulation features with food type (tempting vs. neutral) as the independent variable. Supporting the hypothesis, a main effect of food type occurred, *F*_(1,32)_ = 171.20, *p* < 0.001, $\eta_{p}^{2}$ =0.84. More than twice the proportion of many eating simulation features was generated for tempting foods (*M* = 53%, SE = 2%) than for neutral foods (*M* = 26%, SE = 2%). The dark gray bars in **Figure 1** display this difference, with the light gray bars in **Figure 1** displaying the corresponding percentages of all other types of features for tempting foods (*M* = 47%, SE = 2%) and neutral foods (*M* = 74%, SE = 2%).

### DETAILED ANALYSIS OF EATING SIMULATION FEATURES

**FIGURE 2:**
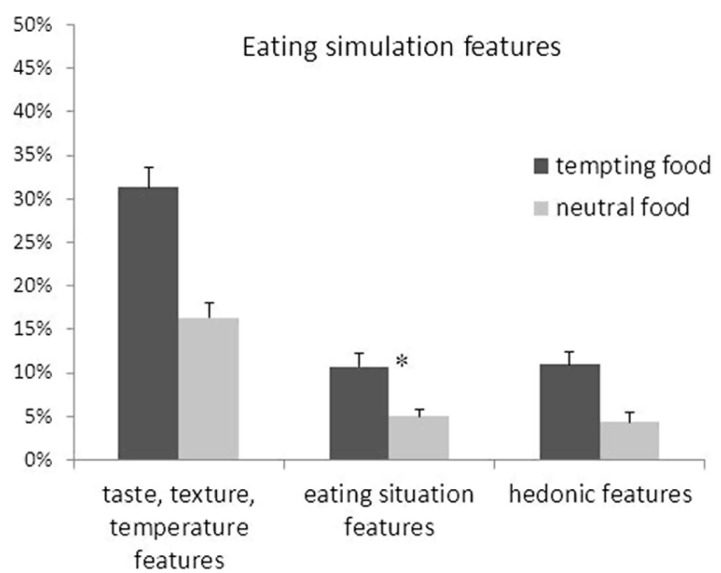
**Percentages of each of three types of eating simulation features generated for tempting and neutral food. Error bars denote the standard error of the mean.** An asterisk marks a significant difference between tempting and neutral food (*p* < 0.001).

To examine in more detail on which of the eating simulation features the food types differ, tempting and neutral food were compared on each of the three specific types of eating stimulation features. To this end, a repeated measures ANOVA was conducted with food type (tempting vs. neutral) and specific feature type (taste/texture/temperature vs. eating situation vs. hedonic) as independent variables. As can be seen in **Figure 2**, this analysis revealed a main effect of feature type, *F*_(2,31)_ = 29.77, *p* < 0.001, $\eta_{p}^{2}$ = 0.66, as well as an interaction of food and feature type, *F*_(2,31)_ = 4.54, *p* = 0.02, $\eta_{p}^{2}$ =0.23. Simple main effects were then analyzed to examine this interaction further. Specifically, the effect of food type was analyzed for gustatory, hedonic, and situation features separately. These analyses showed that the difference between tempting and neutral food was slightly larger for taste, texture, and temperature features, *F*_(1,32)_ = 40.38, *p* < 0.001, $\eta_{p}^{2}$ =0.56, and hedonic features, *F*_(1,32)_ = 40.28, *p* < 0.001, $\eta_{p}^{2}$ =0.56, compared to situation features, *F*_(1,32)_ = 24.89, *p* < 0.001, $\eta_{p}^{2}$ =0.44. All contrasts, however, were highly significant. This set of findings clearly shows that participants were more likely to mention taste, texture and temperature, eating situations, and hedonic experiences when describing tempting food compared to neutral food.

### DETAILED ANALYSIS OF OTHER TYPES OF FEATURES

**FIGURE 3:**
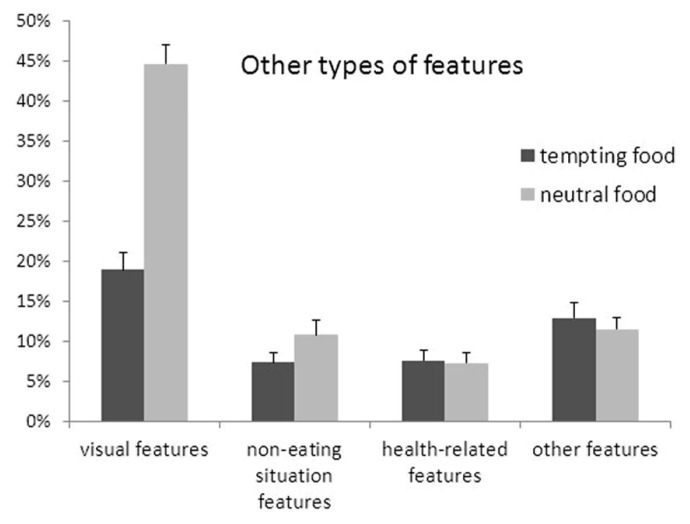
**Percentages of each of four types of other features generated for tempting and neutral food.** Error bars denote the standard error of the mean. An asterisk marks a significant difference between tempting and neutral food (*p* < 0.001), and a “^†^” marks a marginally significant difference (*p* < 0.10).

To examine whether tempting and neutral food also differ on the proportions of other specific feature types, a repeated measures ANOVA was conducted with food type (tempting vs. neutral) and feature type (visual vs. non-eating situation vs. health vs. other) as independent variables. **Figure 3** displays the results of this test. Again, a main effect of feature type, *F*_(3,30)_ = 28.50, *p* < 0.001, $\eta_{p}^{2}$ =0.74, was qualified by an interaction with food type, *F*_(3,30)_ = 26.38, *p* < 0.001, $\eta_{p}^{2}$ =0.73. Contrast analyses showed that only the number of visual features differed between tempting and neutral food, *F*_(1,32)_ = 118.58, *p* < 0.001, $\eta_{p}^{2}$ =0.79, such that more visual features were generated for neutral (*M* = 45%, SE = 2%) than for tempting food (*M* = 19%, SE = 2%). For non-eating situation features, there was a marginal effect of food type, *F*_(1,32)_ = 3.95, *p* = 0.06, $\eta_{p}^{2}$ =0.11, such that more non-eating situation features were generated for neutral (*M* = 11%, SE = 2%) than for tempting food (*M* = 7%, SE = 1%). With regard to the health and other features, there were no differences between tempting and neutral food, both *p* > 0.29.

This set of findings shows that participants were more likely to mention visual features when describing neutral food compared to tempting food, and somewhat more likely to mention non-eating situation features. At the same time, there were no differences between tempting and neutral food in the degree to which participants described them in terms of health and other types of information.

### PROFILES OF TEMPTING AND NEUTRAL FOOD

**FIGURE 4:**
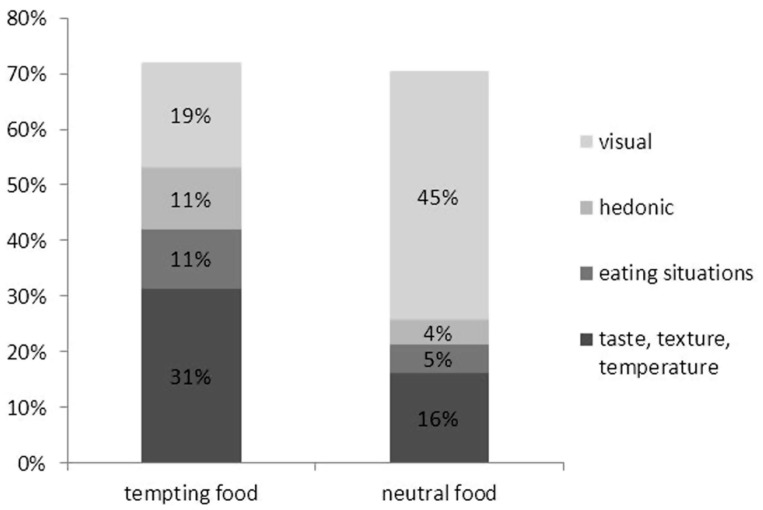
**Percentages of eating simulation and visual features, constituting different profiles for tempting and neutral food**.

The previous analyses suggest that different types of information play a role in the representation of tempting and of neutral food items. To integrate these findings into a profile for each food type, **Figure 4** displays the proportions of only those features on which tempting and neutral foods differ significantly (taste, texture, and temperature features, eating situation features, hedonic features, and visual features). The figure clearly shows how tempting and neutral food representations are composed of these features to different degrees. While these four types of features together constitute similar percentages of generated features for tempting and neutral food (72 and 70%, respectively), the proportions of specific feature types differ considerably. We can see in the figure that tempting food is mostly represented with features for eating simulations (53%), most notably taste, texture, and temperature features (31%), whereas neutral food is mostly represented in terms of visual features (45%).

This pattern was further corroborated by an analysis of the first features of each feature list. Specifically, it was counted whether an individual feature list produced by a participant started with an eating simulation feature, or with a visual feature. This analysis found that of all feature lists produced in response to the tempting food words, 59.8% started with an eating simulation feature, whereas 24.2% started with a visual feature. In contrast, 70.5% of feature lists for neutral foods started with a visual feature, and 9.1% started with an eating simulation feature.

### MANIPULATION CHECK ON HEDONIC AND HEALTH FEATURES

In a final set of analyses, subcategories of health and hedonic features were examined to perform a manipulation check for the categorization of the food items as *tempting* (i.e., highly attractive but unhealthy) and *neutral* (i.e., less attractive but healthy). To this end, positive (“tasty”) and negative (“disgusting”) hedonic features were now analyzed as separate categories, as were positive (“healthy”) and negative (“makes you fat”) health features.

#### Positive and negative hedonic features

First, hedonic features were analyzed in a repeated measures ANOVA with food type (tempting vs. neutral) and hedonic feature valence (positive vs. negative) as independent variables. As expected, participants generated more positive hedonic features for tempting food (*M* = 10%, SE = 1%) than for neutral food (*M* = 4%, SE = 0.1%), *F*_(1,32)_ = 42.61, *p* < 0.001, $\eta_{p}^{2}$ =0.57. There was no difference in the percentage of negative hedonic features generated for tempting and for neutral food, although the difference was in the predicted direction (*M* = 0.1 and 0.7%, respectively), *p* > 0.3. Because the neutral foods were actually not unattractive, it makes sense that negative hedonic features were not produced for them frequently. Overall, the interaction of food type and hedonic feature valence was highly significant, *F*_(1,32)_ = 30.42, *p* < 0.001, $\eta_{p}^{2}$ =0.49. Thus, participants spontaneously described the tempting food as tasty, and did not do so for the neutral food.

In addition, overall more hedonic features were generated for tempting compared to neutral food, as we saw above, *F*_(1,32)_ = 40.28, *p* < 0.001, $\eta_{p}^{2}$ =0.56, and overall more positive than negative hedonic features were generated, *F*_(1,32)_ = 51.93, *p* < 0.001, $\eta_{p}^{2}$ =0.62.

#### Positive and negative health features

Next, health-related features were analyzed similarly in a food type (tempting vs. neutral) and health feature valence (positive vs. negative) repeated measures ANOVA. As expected, participants generated more negative health features for tempting food (*M* = 8%, SE = 1%) than for neutral food (*M* = 0.2%, SE = 0.2%), *F*_(1,32)_ = 36.18, *p* < 0.001, $\eta_{p}^{2}$ =0.53. In contrast, participants generated more positive health features for neutral food (*M* = 7%, SE = 1% ) than for tempting food (*M* = 0.00), *F*_(1,32)_ = 30.68, *p* < 0.001, $\eta_{p}^{2}$ =0.49. As a result, the interaction of food type and health feature valence was highly significant, *F*_(1,32)_ = 43.26, *p* < 0.001, $\eta_{p}^{2}$ =0.58. In sum, participants spontaneously described the tempting food as unhealthy, and the neutral food as healthy.

Together, these findings on hedonic and health features support the categorization of the food items as *tempting* (i.e., highly attractive but unhealthy) and *neutral* (i.e., less attractive but healthy).

### EXPLORING CORRELATIONS WITH EATING SIMULATIONS

Finally, an exploratory correlation analysis was conducted to examine potential associations of eating simulations with individual difference measures in eating and dieting motivation. The following variables were included: proportions of eating simulations for tempting food and for neutral food, averaged tastiness ratings for tempting and for neutral food, averaged current desire to eat tempting and neutral food, hunger, BMI, and chronic dieting as assessed with the restraint scale (Herman and Polivy, 1980; Stroebe et al., 2013), as well as the importance of dieting and being slim. This analysis revealed that the percentage of eating simulation features for tempting food was somewhat negatively associated with chronic dieting, *r*_(33)_ = -0.34, *p* = 0.05, suggesting that chronic dieters produced fewer eating simulation features than non-dieters. This correlation was absent for neutral foods, *p* = 0.78.

In addition, the percentage of eating simulation features was positively associated with explicit tastiness ratings, *r*_(33)_ = 0.37, *p* = 0.04, and there was a trend in the same direction for the current desire to eat these items, *r*_(33)_ = 0.27, *p* = 0.13. Again, these correlations were largely absent for neutral food, *r*_(33)_ = 0.12, *p* = 0.50 and *r*_(33)_ = 0.25, *p* = 0.17. Although they should be interpreted with caution, these findings suggest that eating simulations are associated with the perceived goal relevance of the food items, particularly when it comes to tempting food. Future studies with larger samples could examine systematically which individual differences in eating and dieting motivation are related to eating simulations.

## DISCUSSION

This study examined the role of eating simulations in the representation of food. The findings suggest that food words activate eating simulations, particularly when these words refer to tempting (i.e., attractive, unhealthy) food objects. When asked to name features of foods, 53% of the features that participants listed for tempting foods referred to eating simulations, compared to 26% of the features listed for neutral foods. In contrast, visual features dominated the feature lists for neutral foods (45%) but were much less salient for tempting foods (19%). In addition, the feature lists that participants produced for tempting foods typically started with eating simulation feature, whereas the lists for neutral foods mostly started with a visual feature. In other words, tempting foods, such as *chips*, were spontaneously described in terms of what it is like to eat them, whereas neutral foods, such as *rice*, were described in terms of what they look like.

Thus, eating simulations seem to be an inherent part of our knowledge about food, with this being particularly true for attractive, unhealthy food. These findings are in line with the growing literature on grounded cognition, which has shown that simulations of perceptual experiences are important in conceptual knowledge more generally. However, no earlier work has specifically applied this perspective to the representation of food. This initial study explored the features that participants generate when they list features for food words in a relatively unconstrained manner, and these features were coded as referring to parts of an eating simulation or not. This approach might prove to be very informative as to how individuals think about different types of food, and how their representations of food are grounded in their individual motivation and eating experiences.

### THE CONTENT OF FOOD REPRESENTATIONS

As can be seen in the feature profiles for tempting and neutral food that participants generated (**Figure 4**), they strongly relied on taste, texture, and temperature features when describing food, particularly tempting food. These aspects thus seem to be particularly salient for such foods. In fact, one might argue that they might be so salient as to have become linguistically associated, so that they can be retrieved from memory via word associations, and without relying on an actual eating simulation (see Santos et al., 2011). However, research on word association does not show that taste and texture features are strongly associated with tempting food words. A brief inspection of free association norms suggest that the strongest word associations for chips, for example, are “dip,” “potato,” and “food,” for *cookie*, these are “chocolate,” “jar,” and “monster,” and for ice cream, these are “cold,” “cone,” and “chocolate” (Nelson et al., 2004). None of these are clearly taste or texture features, and only “cold” would be treated as an eating simulation feature in the current coding scheme. These differences suggest that the features that participants produced in the current study are not mainly the result of word association. Nevertheless, future research might try to systematically disentangle whether features generated for food words are the result of eating simulations, word associations, or result from a mixture of both processes.

Results also showed that participants made much use of hedonic features, especially for tempting food, suggesting that the hedonic experience associated with eating these foods is an important part of how people represent them. This finding is consistent with much research showing that tasty, high-calorie foods are experienced as rewarding (e.g., Drewnowski, 1995; Pinel et al., 2000), despite the unfavorable health consequences of (over)consuming them. Similarly, such foods have been found to activate positive affect in studies assessing implicit attitudes toward food (Hoefling and Strack, 2008; Papies et al., 2009) and to activate reward-processing areas in the brain (e.g., Simmons et al., 2005; van der Laan et al., 2011), which also is consistent with the current finding that this food is often described spontaneously by referring to its reward value.

Interestingly, participants often listed features of eating situations, without being asked, and again did so more often for tempting than for neutral food. Strictly speaking, these features are not directly properties of the food objects, and are therefore not requested from participants, who were clearly instructed to list “properties that are typically true of an object.” However, participants listed words such as “birthday,” “sofa,” or “movie” for the concept *chips*, and responded with “beer,” “end of afternoon,” or “bowl” when asked to provide features of *cocktail nuts*. Clearly, participants do not mean to imply that “sofa” is directly a physical property of *chips*, but they seem to construct a simulation of a situation in which chips are eaten, and proceed to describes features of that situation, as if they were features of the food itself (given that they were asked to generate features of the food). Thus, eating situations indeed seem to be a relevant part of our representation of foods. Even though these features are not part of the foods themselves, they describe central aspects of consuming them. In this regard, food representations seem more “liberal” and inclusive than one might assume initially (see also Keller and van der Horst, 2013).

In this context, it is also interesting to note that there was a high degree of variation in the eating situation features generated by participants, particularly for tempting foods (see **Table 1**). Specifically, looking at the third feature column in **Table 1**, where eating situation features for the tempting food are listed, we can see that long lists of context cues are provided by participants for each tempting food word. The number and variety of situation features is particularly striking when compared to the columns listing hedonic features, where often only one or two different words are provided. The richness of the situation features suggests that the specific instances of the food items that participants were retrieving during the task were highly idiosyncratic, which may reflect the fact that participants were heavily relying on specific earlier experiences with the foods. These findings again support a simulation perspective of the representation of food, and are in line with findings showing that our simulations of concepts are heavily situated more generally (for a review, see for example Yeh and Barsalou, 2006).

### ASSOCIATIONS WITH MOTIVATION

Two interesting correlational findings suggest that participants’ food representations are modulated not only by their earlier experiences, but also by their motivational states (see also Papies et al., under review). Specifically, exploratory analyses pointed toward the percentage of eating simulations for tempting foods having a negative association with participants’ chronic dieting concerns, and a positive association with attractiveness ratings. In other words, chronic dieters listed fewer eating simulation features of tempting food than non-dieters, and participants who liked these foods more listed more eating simulation features for them than participants who like these foods less. Although the feature listing task allows participants to consciously control their answers, it seems unlikely that these effects are due to social desirability concerns. Specifically, participants most likely were not aware that we were interested in the proportions of taste, texture, temperature, hedonic, and eating situation features they provided. Rather, these findings support the perspective that food representations indeed contain information that is relevant for goal-directed behavior: depending on whether eating the food is a desirable goal for the individual or not, eating simulations are generated to a greater or lesser degree. This is consistent with earlier work showing that chronic dieters are ambivalent about tempting food and evaluate it less positively than non-dieters, because it interferes with their dieting goals (e.g., Hoefling and Strack, 2008; Stroebe et al., 2008; Papies et al., 2009; Keller and van der Horst, 2013). The current findings suggest that chronic dieters also simulate eating such foods to a lesser degree, which might be beneficial for their dieting behavior. Future research could examine in more detail which individual differences are associated with eating simulations, and how this in turn modulates behavior.

If food is indeed represented by eating simulations, how do these simulations function to affect consummatory behavior? Barsalou (2009) suggested that when we encounter a cue that is part of a situated simulation that we have stored from previous experiences, pattern completion inferences may activate other components of the representation. As a result, we can predict future events, including possible actions and their effects. Applying this mechanism here suggests that when some aspect of an previous eating experience (e.g., the salty taste of chips) is re-activated by a relevant cue (e.g., seeing a bag of chips in a store), a pattern completion mechanism may activate reward features that have been previously encoded in these same eating experiences (Papies and Barsalou, in preparation), as well as the representation of relevant actions for obtaining that reward. In other words, pattern completion inferences involving reward signals trigger goal-directed behavior toward obtaining the food. Importantly, while this process can be triggered by conscious mental imagery (see Kavanagh et al., 2005), it could also be activated more automatically and guide behavior without conscious awareness. The current findings suggest that such simulations of interacting with the object are indeed represented within situated simulations of the food objects, so that they can inform and guide relevant behavior without conscious awareness or elaboration.

Interestingly, this implies that via a pattern completion mechanism, situational cues themselves (e.g., “watching a movie”) could activate the rest of a situated eating simulation of a food that one often eats in such situations (“chips”), including the taste, texture, and reward cues, and therefore motivate the purchase and consumption of the food. In other words, because of the situated nature of our representations of food, a seemingly unrelated contextual cue, such as someone mentioning one’s favorite movie, could activate the re-experience of eating crunchy paprika chips on the sofa, and thus trigger motivated behavior to create that experience. This way, effects on behavior could occur even without conscious awareness or elaboration, or without the actual experience of a craving. In fact, recent work on habits has shown that contextual cues can trigger behavior in such ways (see Neal et al., 2006). An intriguing possibility is that eating habits are represented in terms of the situated eating simulations observed here. The precise nature and internal structure of these representations, and their links with motivated behavior, need to be examined in more detail.

### IMPLICATIONS FOR INTERVENTIONS

Even though more work is clearly needed on this topic, realizing that eating simulations are an inherent part of food representations (especially for tempting food) already has important implications for interventions to increase healthy eating. One example is the class of interventions designed to change the reward value of a food (e.g., Hollands et al., 2011; Havermans, 2013). The present work implies that these interventions might benefit from targeting not only the food object itself, but the reward of the complete eating situation, as this is likely to be simulated when the food is encountered. This points to another important implication for interventions, namely, the potentially highly idiosyncratic, situated nature of food representations. Again, however, more research should establish this in more detail, and perhaps develop novel ways of assessing individual variations in food representations systematically.

While this work provided important initial insights into the nature of food representations and the role of eating simulations, it also has a number of limitations that might be avoided in future research. One limitation of this small-scale study is that only four food words of each type were used, making it difficult to generalize to healthy and unhealthy food categories more broadly. Future work should examine to what degree the effects reported here are more generally true for these food categories, rather than for the specific items that were used in this study.

In addition, although feature listing is a technique that allows the researcher to collect rich data on how participants idiosyncratically represent food and eating contexts, it only provides a small window into cognitive representations. Some types of information are more difficult to verbalize than others, so that they may be underrepresented in the features listed (see McRae et al., 2005). In the present research, this may be particularly true for taste and texture features, as these are experienced in great detail, but difficult to convey linguistically (e.g., Melcher and Schooler, 1996). Although this might have restricted participants here, taste and texture features still constituted a large percentage of the features mentioned for food items. This suggests that they are an important conscious aspect of the simulations that food words activate, although we may not have many different words for them. For this reason, future work might develop further methods that go beyond feature listing to examine the complex representations of food even more deeply.

## Conflict of Interest Statement

The authors declare that the research was conducted in the absence of any commercial or financial relationships that could be construed as a potential conflict of interest.

## Footnotes

1 An additional 33 participants participated in a different condition of the experiment and were chewing gum during the feature listing task. This manipulation was included to explore the hypothesis that it would reduce the number of eating simulations, which was not supported. Because it cannot be ruled out that the manipulation did affect other processes potentially relevant for the current study, only the findings of the control condition are reported here.">An additional 33 participants participated in a different condition of the experiment and were chewing gum during the feature listing task. This manipulation was included to explore the hypothesis that it would reduce the number of eating simulations, which was not supported. Because it cannot be ruled out that the manipulation did affect other processes potentially relevant for the current study, only the findings of the control condition are reported here.
